# A Plant-Based Strategy for MASLD: *Desmodium caudatum* (Thunb.) DC. Extract Reduces Hepatic Lipid Accumulation and Improves Glycogen Storage In Vitro and In Vivo

**DOI:** 10.3390/ijms26178442

**Published:** 2025-08-30

**Authors:** Yu-Ching Chen, Yu-Hsuan Liang, Yueching Wong, Chiao-Yun Tseng, Chi-Wen Chang, Hui-Hsuan Lin, Jing-Hsien Chen

**Affiliations:** 1 Department of Endocrinology and Metabolism, Antai Medical Care Corporation Antai Tian-Sheng Memorial Hospital, Pingtung 928, Taiwan; veronica5403@gmail.com; 2 Department of Nutrition, Chung Shan Medical University, Taichung City 402, Taiwan; 1346003@live.csmu.edu.tw (Y.-H.L.); wyc@csmu.edu.tw (Y.W.); 1146002@live.csmu.edu.tw (C.-Y.T.); zx2653186@gmail.com (C.-W.C.); 3 Department of Medical Laboratory and Biotechnology, Chung Shan Medical University, Taichung City 402, Taiwan; linhh@csmu.edu.tw; 4 Department of Medical Research, Chung Shan Medical University Hospital, Taichung City 402, Taiwan

**Keywords:** metabolic dysfunction-associated steatotic liver disease, *Desmodium caudatum* (Thunb.) DC., lipid accumulation, glycogen metabolism

## Abstract

Metabolic dysfunction-associated steatotic liver disease (MASLD) is characterized by hepatic lipid accumulation and insulin resistance, yet effective therapies remain limited. This study evaluated the hepatoprotective effects of *Desmodium caudatum* (Thunb.) DC. Extract (DCE) in vitro and in vivo. In 600 μM oleic acid (OA)-challenged HepG2 cells, DCE (25, 50, and 100 μg/mL) reduced lipid accumulation, oxidative stress, and glycogen depletion by modulating lipogenic and oxidative pathways. In a MASLD mouse model induced by high-fat diet (HFD)/streptozotocin (HFD/STZ), oral administration of DCE (100 or 200 mg/kg) for six weeks improved fasting glucose, serum lipids, and hepatic injury markers. Histology confirmed reduced steatosis, while Western blotting showed downregulation of SREBP-1, HMGCR, and ACC, and upregulation of CPT-1, PPARα, and phosphorylated AMPK. Additionally, DCE enhanced insulin signaling and restored hepatic glycogen synthesis through IRS-1, AKT, and GSK3β activation. These findings suggest that DCE ameliorates MASLD by regulating lipid and glucose metabolism, supporting its potential as a plant-based therapeutic strategy.

## 1. Introduction

Metabolic dysfunction-associated steatotic liver disease (MASLD) has rapidly ascended to the forefront of global chronic liver diseases, posing a significant public health challenge. The defining characteristic of MASLD is the excessive accumulation of lipids within the liver parenchyma, a condition termed hepatocyte steatosis [1]. Untreated steatosis can progress to metabolic dysfunction-associated steatohepatitis (MASH), potentially leading to fibrosis, cirrhosis, and hepatocellular carcinoma [2], underscoring the critical need for effective interventions. Epidemiological data indicate that the prevalence of MASLD has now surpassed that of both viral hepatitis and alcoholic liver disease (ALD) [3]. In Western populations, MASLD affects an estimated 20–30% of adults, and, globally, nearly one billion individuals are thought to be affected, with prevalence rates continuing to rise [4]. Despite ongoing clinical trials, there remains a significant void in approved pharmacological treatments specifically targeting MASLD [5]. Currently, clinical management often relies on addressing underlying metabolic imbalances using agents like statins and metformin, which can offer some benefits in the context of MASLD.

At the core of MASLD pathogenesis lies the dysregulation of hepatic lipid metabolism. Lipid accumulation occurs when the rate of lipid synthesis exceeds fatty acid oxidation, leading to the pathological deposition of lipids within the liver [6]. De novo cholesterol synthesis is controlled by the enzyme hydroxymethylglutaryl-CoA reductase (HMGCR) and is upregulated by the sterol regulatory element-binding protein 2 (SREBP-2) [7,8]. Meanwhile, triglyceride (TG) synthesis is regulated by acetyl-CoA carboxylase (ACC) and fatty acid synthase (FAS) [9]. Conversely, the efficient removal of fatty acids through β-oxidation is crucial for maintaining lipid homeostasis. Carnitine palmitoyltransferase I (CPT-1) is a key enzyme in this process, and its expression is positively regulated by peroxisome proliferator-activated receptor α (PPARα). Notably, PPARα activation enhances β-oxidation and suppresses the expression of SREBP-1, ACC, and FAS, thus providing a dual mechanism for reducing hepatic lipid content [10,11]. The AMP-activated protein kinase (AMPK) is a critical cellular energy sensor, playing a multifaceted role in regulating lipid and glucose metabolism. Upon activation via phosphorylation, AMPK inhibits ACC and HMGCR, thereby reducing cholesterol and fatty acid synthesis [12]. Furthermore, AMPK enhances CPT-1 activity, promoting fatty acid oxidation. Beyond lipid metabolism, AMPK also modulates glucose metabolism by promoting glycogen synthesis and improving insulin sensitivity, collectively contributing to alleviating MASLD-related metabolic disturbances [13].

Excessive hepatic TG accumulation can also lead to the development of insulin resistance, reducing insulin sensitivity and contributing to metabolic disorders such as diabetes, metabolic syndrome, and MASLD. Defects in insulin signaling pathways are a major underlying cause, with insulin receptor substrate 1 (IRS-1) and insulin receptor substrate 2 (IRS-2) playing pivotal roles in mediating insulin’s effects on glucose metabolism [14]. Tyrosine (Tyr) phosphorylation of IRS-1 typically promotes downstream insulin signaling through the activation of phosphoinositide 3-kinase (PI3K) and serine/threonine kinase (AKT). However, chronic hyperinsulinemia can paradoxically lead to serine (Ser) phosphorylation of IRS-1, which inhibits its function and impairs insulin signaling [15]. AKT (protein kinase B, PKB), a downstream effector of PI3K, phosphorylates glycogen synthase kinase-3β (GSK3β), leading to its inactivation and the subsequent activation of glycogen synthase (GS), enhancing glycogen synthesis, reducing hepatic glucose production, and maintaining glycogen storage [16]. Metformin, a commonly used drug for type 2 diabetes, improves insulin sensitivity by reducing hepatic gluconeogenesis and enhancing glucose uptake in peripheral tissues [17], highlighting the interconnectedness of glucose and lipid metabolism in MASLD.

*Desmodium caudatum* (Thunb.) DC., a small leguminous tree with a wide distribution across China, India, Japan, and the Philippines, has a rich history of use in Traditional Chinese Medicine (TCM). Traditionally, it has been employed to address a range of conditions, including relieving internal heat or fever, neutralizing toxins, inhibiting pain, invigorating blood circulation, suppressing cough, and alleviating dyspnea [18]. Previous research has revealed that the ethanol extract of the entire plant possesses diverse biological properties, including analgesic, anti-inflammatory, and anti-cancer potential in animal models [19]. TCM has a long-standing and extensive history in managing hepatic diseases, especially in MASLD [20]. The TCM approach to hepatoprotection often emphasizes a holistic perspective, targeting multiple pathological mechanisms simultaneously. These mechanisms include mitigating oxidative stress, modulating lipid metabolism, and preventing the progression of liver fibrosis [20]. Despite the growing interest in and application of TCM in liver disease management, the specific effects of aqueous extracts of *Desmodium caudatum* (Thunb.) DC. On the complex pathophysiology of MASLD has, to our knowledge, not been previously investigated. Therefore, this study aims to examine whether *Desmodium caudatum* (Thunb.) DC. Has hepatoprotective effects, particularly by examining its impact on hepatic lipid accumulation and related metabolic pathways implicated in the development and progression of MASLD.

## 2. Results

### 2.1. DCE Attenuates OA-Induced Cytotoxicity and Oxidative Stress in HepG2 Cells

**Figure 1:**
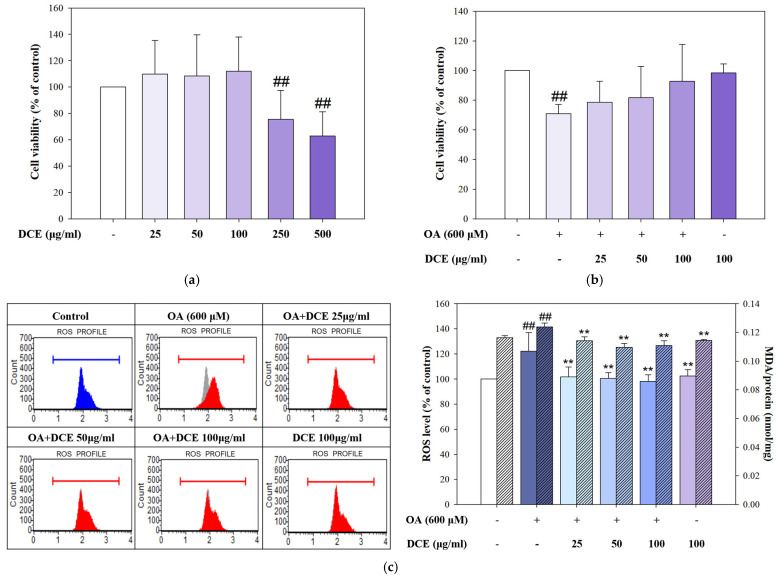
DCE attenuates OA−induced cytotoxicity and oxidative stress in HepG2 cells. (**a**) HepG2 cells were treated with various concentrations of DCE (−25, 50, 100, 250, and 500 μg/mL) for 24 h. (**b**) HepG2 cells were co-treated with OA (600 μM) and different concentrations of DCE (25, 50, and 100 μg/mL) for 24 h, and cell viability was assessed. (**c**) The ROS levels were determined by DCF-DA staining with flow cytometry (control is shown as an overlay in the gray area) and are presented in the **left** panel, while lipid peroxidation was assessed using the TBARS assay and is shown in the **right** panel. Quantitative data are presented as the mean ± SD (n = 3). ^##^ *p* < 0.01 comparison to the control group. ** p < 0.01 compared with the OA-treated group. DCE, *Desmodium caudatum* (Thunb.) DC. extract. OA, oleic acid.

To evaluate the cytotoxic profile of DCE, HepG2 cells were initially treated with increasing concentrations of DCE alone (25–500 μg/mL). As shown in Figure 1a, treatment with DCE at concentrations up to 100 μg/mL did not significantly affect cell viability, whereas exposure to 250 and 500 μg/mL resulted in a marked reduction in cell survival, indicating potential cytotoxicity at higher doses. To assess the cytoprotective potential of DCE, HepG2 cells were co-treated with 600 μM oleic acid (OA) and varying concentrations of DCE (25–100 μg/mL). OA significantly reduced cell viability, whereas co-treatment with DCE effectively restored cell viability in a dose-dependent manner, suggesting that DCE mitigates OA-induced lipotoxicity (Figure 1b). Oxidative stress plays a critical role in the progression from hyperlipidemia to MASLD. Flow cytometric analysis investigated whether DCE could protect HepG2 cells by reducing OA-induced intracellular ROS production. OA treatment led to a substantial elevation in ROS production compared to control cells, whereas co-treatment with DCE markedly reduced ROS accumulation (Figure 1c). In parallel, lipid peroxidation, as measured by TBARS assay (detection of MDA level), was also significantly increased following OA treatment, but was dose-dependently suppressed by DCE. These findings indicate that DCE protects against OA-induced cell death and alleviates oxidative stress and lipid peroxidation in HepG2 cells.

### 2.2. DCE Reduced OA-Induced Lipid Accumulation in HepG2 Cells

**Figure 2:**
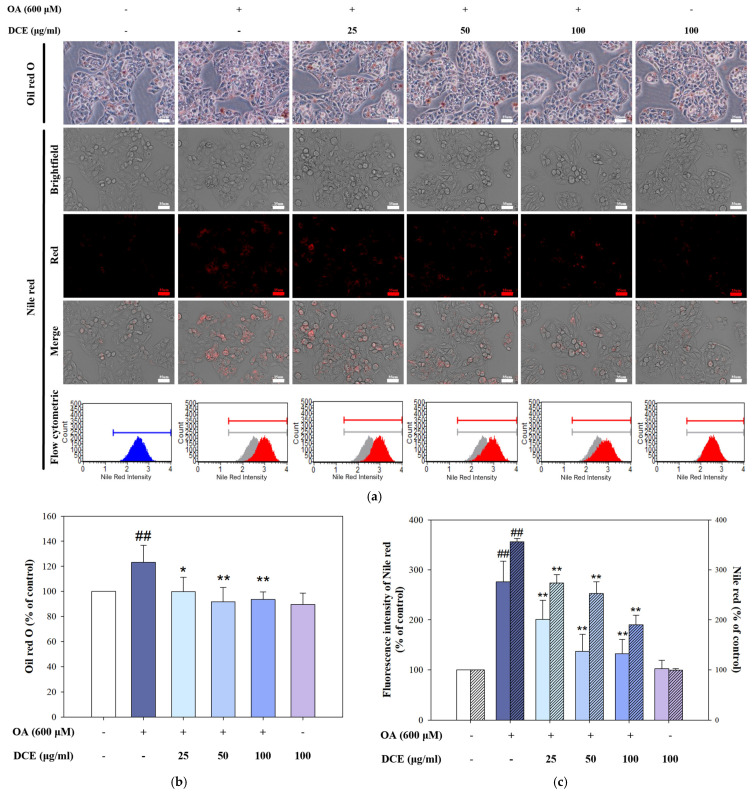
DCE reduced OA−induced lipid accumulation in HepG2 cells. HepG2 cells were co-treated with OA (600 μM) and DCE (25, 50, and 100 μg/mL) for 24 h. (**a**) Representative photomicrographs of HepG2 cells stained with Oil red O (upper panel) and Nile red (lower panel) showed lipid droplet accumulation (red staining) and the intracellular fat content by flow cytometric analysis. Quantitative data of Oil red O (**left** panel) (**b**) and Nile red (**right** panel) (**c**) are presented as the mean ± SD (n = 3). ^##^ *p* < 0.01 compared to the control group. * *p* < 0.05, ** p < 0.01 compared with the OA-treated group. DCE, *Desmodium caudatum* (Thunb.) DC. extract. OA, oleic acid.

To investigate whether DCE mitigates OA-induced lipid accumulation, HepG2 cells were treated with 600 μM OA in the presence or absence of DCE (25, 50, or 100 μg/mL) for 24 h, followed by Oil red O and Nile red staining. In Figure 2a (upper panel: Oil red O; lower panel: Nile red), OA exposure markedly increased intracellular lipid droplet formation, as evidenced by enhanced Oil red O staining and elevated Nile red fluorescence. Co-treatment with DCE resulted in a noticeable reduction in lipid accumulation. Quantification of Oil red O staining confirmed that DCE significantly suppressed OA-induced lipid storage in a concentration-dependent manner (Figure 2b). Likewise, Nile red fluorescence intensity, reflecting neutral lipid content, was substantially decreased in DCE-treated cells relative to the OA alone group (Figure 2c). These results were further supported by flow cytometric analysis, which demonstrated a leftward shift in Nile red fluorescence intensity distribution following DCE co-treatment, indicating a reduction in intracellular lipid burden. These findings suggest that DCE effectively alleviates OA-induced lipid droplet accumulation in HepG2 cells.

### 2.3. DCE Modulates Lipid Metabolism-Related Protein Expression in OA-Induced HepG2 Cells

**Figure 3:**
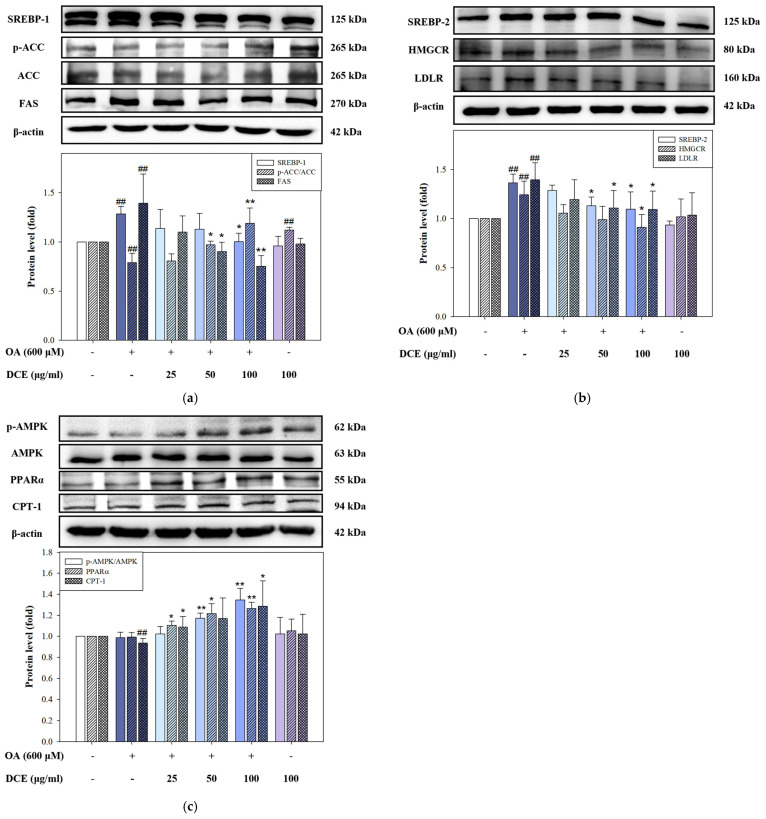
DCE modulates lipid metabolism-related protein expression in OA−induced HepG2 cells. HepG2 cells were co-treated with OA (600 μM) and varying concentrations of DCE (25, 50, and 100 μg/mL) for 24 h. (**a**) The triglyceride synthesis protein levels of SREBP-1, ACC, p-ACC, and FAS. (**b**) The cholesterol synthesis protein levels of SREBP-2, HMGCR, and LDL-R. (**c**) The fatty acid oxidation protein levels of p-AMPK, AMPK, PPARα, and CPT-1 were determined by Western blotting. β-actin served as an internal loading control. Quantitative data are presented as the mean ± SD (n = 3). ^##^ *p* < 0.01 compared to the control group. * *p* < 0.05, ** p < 0.01 compared with the OA-treated group. DCE, *Desmodium caudatum* (Thunb.) DC. extract. OA, oleic acid.

The expression of key regulatory proteins involved in lipogenesis, cholesterol synthesis, and fatty acid oxidation was examined in HepG2 cells treated with OA and DCE to delineate the molecular mechanisms by which DCE attenuates hepatic lipid accumulation. In Figure 3a, OA treatment markedly increased SREBP-1, FAS, and p-ACC protein levels, indicative of activated de novo lipogenesis. Co-treatment with DCE suppressed these lipogenic markers, suggesting an inhibitory effect of DCE on triglyceride synthesis. In the cholesterol synthetic pathway (Figure 3b), OA-induced upregulation of SREBP-2, HMGCR, and LDLR was observed. DCE administration effectively reduced the expression of cholesterol synthesis proteins. Regarding fatty acid catabolism, OA reduced the expression of CPT-1, a rate-limiting enzyme of β-oxidation. At the same time, PPARα and the phosphorylation status of AMPK (a key regulator of lipid metabolism) levels remained unchanged. Notably, DCE restored CPT-1 expression and significantly upregulated the PPARα and p-AMPK/AMPK ratio, suggesting the activation of fatty acid oxidation pathways via AMPK-mediated metabolic reprogramming (Figure 3c). These data demonstrate that DCE counteracts OA-induced dysregulation of lipid metabolism by downregulating lipogenic and cholesterogenic protein expression and enhancing fatty acid oxidation, partially by activating AMPK signaling.

### 2.4. DCE Improves Glycogen Storage by Restoring Insulin Signaling in OA-Challenged HepG2 Cells

**Figure 4:**
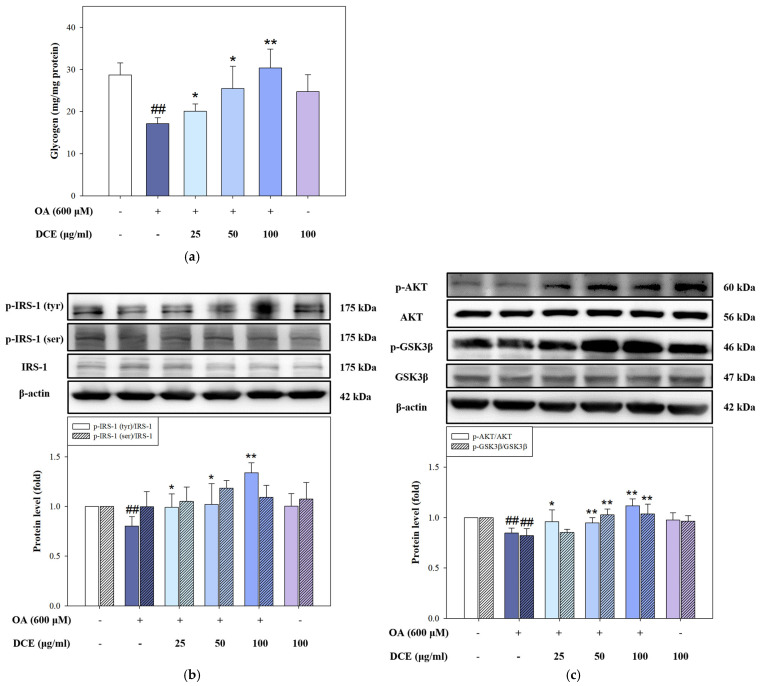
DCE improves glycogen storage by restoring insulin signaling in OA−challenged HepG2 cells. HepG2 cells were co-treated with OA (600 μM) and DCE (25, 50, and 100 μg/mL) for 24 h. (**a**) Intracellular glycogen content was quantified using a Glycogen Assay Kit. The glycogen synthesis protein levels of p-IRS-1 (tyr), p-IRS-1 (ser), IRS-1 (**b**), p-AKT, AKT, p-GSK3β, and GSK3β (**c**) were determined by Western blotting. β-actin served as an internal control. Quantitative data are presented as the mean ± SD (n = 3). ^##^ *p* < 0.01 compared to the control group. * *p* < 0.05, ** p < 0.01 compared with the OA-treated group. DCE, *Desmodium caudatum* (Thunb.) DC. extract. OA, oleic acid.

Excess hepatic lipid burden is frequently accompanied by impaired insulin responsiveness and suppressed glycogen synthesis [21]. To examine whether DCE alleviates these metabolic disturbances, intracellular glycogen levels and key components of the insulin signaling pathway were analyzed in OA-induced HepG2 cells. As shown in Figure 4a, OA exposure significantly decreased glycogen content compared to the control group. Co-treatment with DCE at 25, 50, and 100 μg/mL restored glycogen levels, with the highest recovery observed at 50 and 100 μg/mL, suggesting improved glucose utilization and storage capacity. We assessed the p-IRS-1 at tyrosine (activating) and serine (inhibitory) residues to delineate the signaling events underlying this effect. OA treatment reduced p-IRS-1 (Tyr) and elevated p-IRS-1 (Ser) levels, indicating disrupted insulin signal transduction. DCE administration reversed this pattern, enhancing tyrosine phosphorylation and reducing serine phosphorylation of IRS-1 (Figure 4b), thereby restoring upstream insulin signaling. Further downstream, phosphorylated AKT and GSK3β, key mediators of glycogen synthesis, were significantly suppressed by OA but reinstated in a dose-responsive manner upon DCE co-treatment (Figure 4c). Restoration of AKT and GSK3β phosphorylation implies the reactivation of glycogen synthase activity, facilitating glycogen accumulation. These findings indicate that DCE activates the insulin signaling pathway upstream of glycogen synthase, stabilizing and increasing cellular glycogen storage in OA-induced lipotoxicity.

### 2.5. DCE Improves Hepatic Lipid Accumulation and Serum Biochemical Parameters in HFD/STZ-Induced MASLD Mice

**Figure 5:**
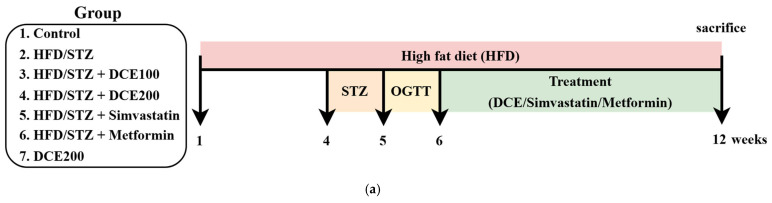

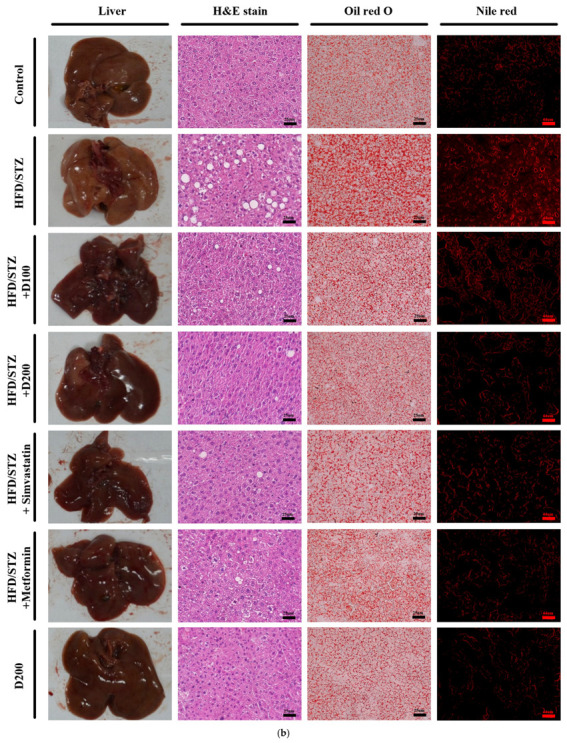

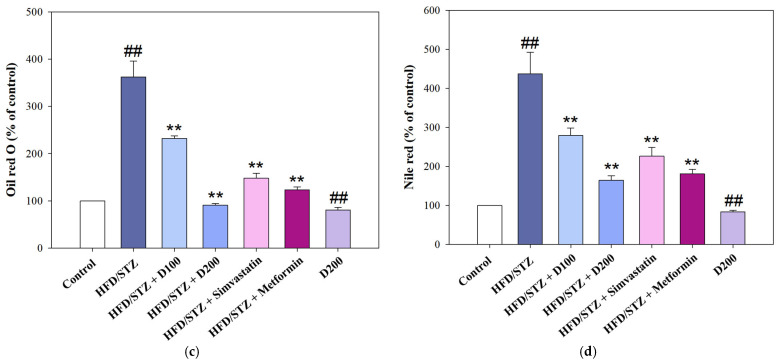
DCE improves hepatic lipid accumulation in HFD/STZ-induced MASLD mice. (**a**) The flowchart of animal experimental design. Mice in the control and DCE-only groups were maintained on a standard commercial chow diet. To induce MASLD, all other groups were fed an HFD for a total duration of 12 weeks. To further mimic metabolic syndrome-associated features, HFD group mice received intraperitoneal STZ injections for five consecutive days during week 4. The establishment of the MASLD model was validated using an oral glucose tolerance test (OGTT) conducted in week 5. Starting from week 6, therapeutic interventions were initiated. Mice in the treatment groups received daily oral gavage of DCE (100 and 200 mg/kg bw), Simvastatin (200 mg/kg bw), and Metformin (250 mg/kg bw) for 6 weeks. All animals were sacrificed at the end of the 12 week experimental period, and blood and liver tissue samples were harvested for subsequent analyses. (**b**) Images of the whole liver tissue and photomicrographs of liver frozen sections stained with H&E, Oil red O, and Nile red. Quantifying lipid accumulation based on Oil red O (**c**) and Nile red (**d**) staining is presented as mean ± SD (n = 3). ^##^ *p* < 0.01 compared with the control group. ** *p* < 0.01 compared with the HFD/STZ-induced group. HFD/STZ and HFD combined with STZ induce MASLD. D100, 100 mg/kg bw *Desmodium caudatum* (Thunb.) DC. extract. D200, 200 mg/kg bw *Desmodium caudatum* (Thunb.) DC. extract. OGTT, oral glucose tolerance test.

To evaluate the therapeutic efficacy of DCE in vivo, a MASLD model was established in C57BL/6 mice using HFD feeding combined with STZ injection. Mice were treated with either DCE (100 or 200 mg/kg), Simvastatin, or Metformin for six weeks (Figure 5a). Body weight tended to increase in the HFD/STZ group compared to the control group; however, the difference did not reach statistical significance (Figure S1a). Additionally, Figure S1b indicates that food intake was comparable across all experimental groups. As presented in Table 1, DCE treatment significantly reduced fasting blood glucose (GLC), total cholesterol (TC), triglycerides (TG), and low-density lipoprotein-cholesterol (LDL-C), while increasing high-density lipoprotein-cholesterol (HDL-C) concentrations. Hepatic injury markers, including aspartate aminotransferase (AST), alanine aminotransferase (ALT), and lactate dehydrogenase (LDH), were all significantly elevated in the HFD/STZ group, but were effectively attenuated by DCE intervention. Histological findings supported these biochemical improvements. Liver morphology revealed pale and enlarged livers in the HFD/STZ group, indicative of steatosis, whereas DCE-, Simvastatin-, and Metformin-treated groups exhibited improved liver appearance. Lipid-specific staining using Oil red O and Nile red demonstrated extensive lipid deposition in the HFD/STZ group. In contrast, DCE administration significantly reduced lipid accumulation dose-dependently (Figure 5b–d). However, the higher dose of DCE (200 mg/kg bw) exhibited a therapeutic effect on hepatic lipid accumulation comparable to that observed with the clinical reference drugs.

### 2.6. The Expression of DCE on Lipid Metabolic Regulators in HFD/STZ-Induced MASLD Mice

**Figure 6:**
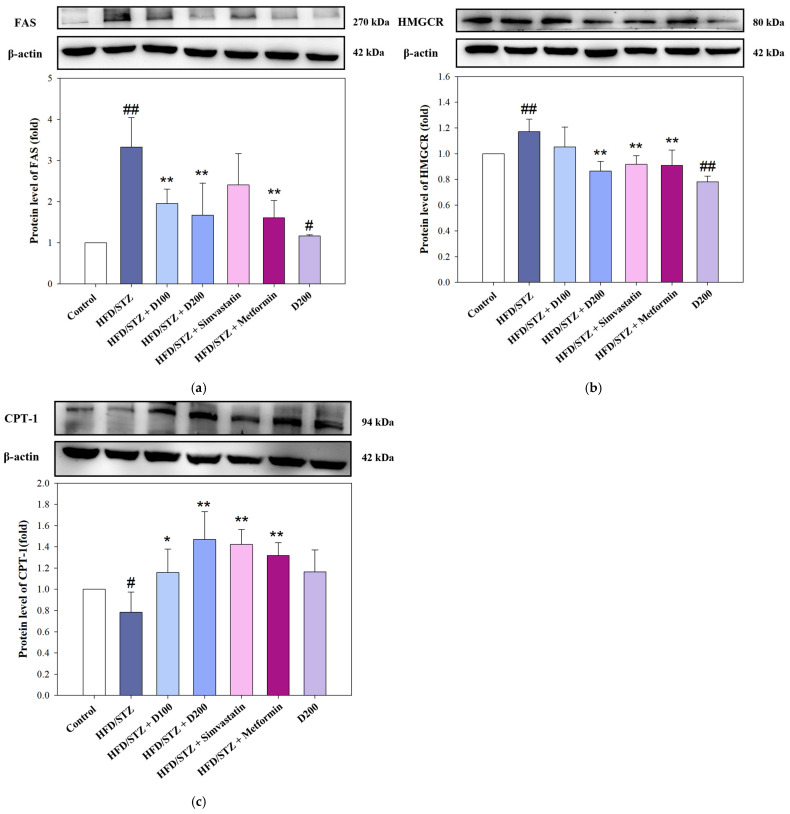
The expression of DCE on lipid metabolic regulators in HFD/STZ-induced MASLD mice. MASLD mice were induced by HFD combined with STZ injection. At the 6th week, mice were treated with DCE (100 and 200 mg/kg bw), Simvastatin (200 mg/kg bw), and Metformin (250 mg/kg bw) for 6 weeks. The mice were sacrificed after 6 weeks, and liver tissue was collected for analysis. (**a**) The triglyceride synthesis protein levels of FAS. (**b**) The cholesterol synthesis protein levels of HMGCR. (**c**) The fatty acid oxidation protein levels of CPT-1 were determined by Western blotting. β-actin served as an internal loading control. Quantitative data are presented as mean ± SD (n = 3). ^#^ *p* < 0.05, ^##^ *p* < 0.01 compared with the control group. * *p* < 0.05, ** *p* < 0.01 compared with the HFD/STZ-induced group. HFD/STZ and HFD combined with STZ induce MASLD. D100, 100 mg/kg bw *Desmodium caudatum* (Thunb.) DC. extract. D200, 200 mg/kg bw *Desmodium caudatum* (Thunb.) DC. extract.

To further elucidate the mechanisms underlying the lipid-lowering effects of DCE, the hepatic expression of key enzymes involved in lipid metabolism was assessed in HFD/STZ-induced MASLD mice. In Figure 6a, FAS, a pivotal enzyme for lipogenesis, was markedly upregulated in the HFD/STZ group. Treatment with DCE significantly suppressed FAS expression, with the higher dose achieving a reduction comparable to metformin. A similar pattern was observed for HMGCR, the rate-limiting enzyme in cholesterol synthesis. DCE markedly attenuated OA-induced HMGCR overexpression, with the 200 mg/kg group exhibiting the most pronounced effect (Figure 6b). DCE administration restored CPT-1 levels, with the 100 and 200 mg/kg groups showing statistically significant increases, like the Simvastatin and Metformin groups (Figure 6c). These results demonstrate that DCE modulates hepatic lipid metabolism by downregulating lipogenic and cholesterogenic pathways, while concurrently enhancing fatty acid oxidation. Among all groups, DCE at 200 mg/kg consistently yielded the most favorable modulation of these lipid-regulatory proteins, supporting its therapeutic potential in managing MASLD-associated dyslipidemia.

### 2.7. The Effect of DCE on Glucose Tolerance and Hepatic Glycogen Synthesis in HFD/STZ-Induced MASLD Mice

**Figure 7:**
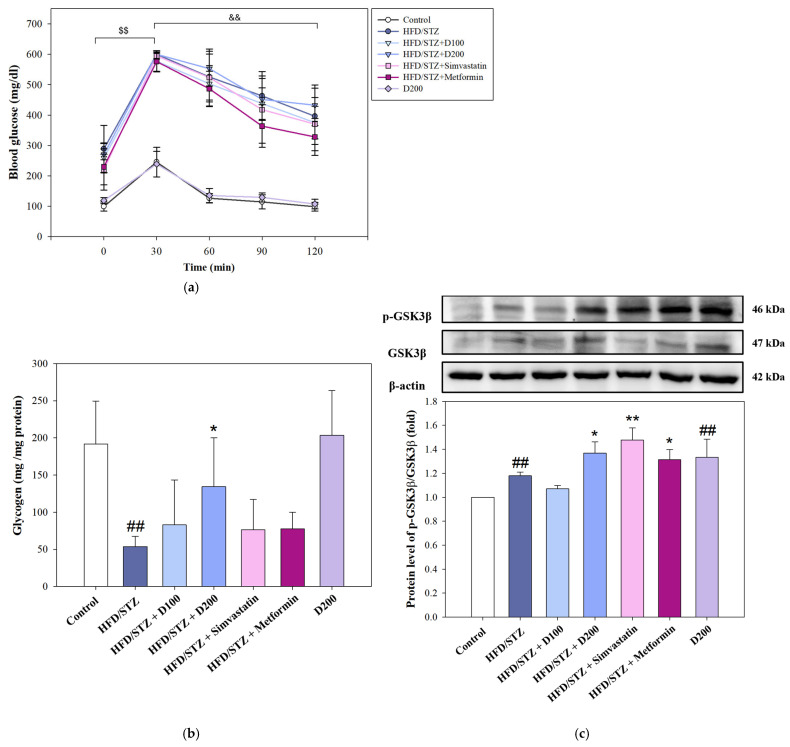
The effect of DCE on glucose tolerance and hepatic glycogen synthesis in HFD/STZ-induced MASLD mice. MASLD mice were induced by HFD combined with STZ injection. At the 6th week, mice were treated with DCE (100 and 200 mg/kg bw), Simvastatin (200 mg/kg bw), and Metformin (250 mg/kg bw) for 6 weeks. The mice were sacrificed after 6 weeks, and liver tissue was collected for analysis. (**a**) Oral glucose tolerance (OGTT) was measured at the 12th week. (**b**) Hepatic glycogen content was detected using a Glycogen Assay Kit. (**c**) The glycogen synthesis protein levels of p-GSK3β and GSK3β were determined by Western blotting. β-actin served as an internal control. Quantitative data are presented as mean ± SD (n = 3). ^$$^ *p* < 0.01 30 min post-load compared to baseline (0 min) within the HFD/STZ group. ^&&^ *p* < 0.01 120 min post-load compared to 30 min. ^##^ *p* < 0.01 compared with the control group. * *p* < 0.05, ** *p* < 0.01 compared with the HFD/STZ-induced group. HFD/STZ and HFD combined with STZ induce MASLD. D100, 100 mg/kg bw *Desmodium caudatum* (Thunb.) DC. extract. D200, 200 mg/kg bw *Desmodium caudatum* (Thunb.) DC. extract.

To assess the impact of DCE on glucose homeostasis, an OGTT was performed following six weeks of treatment. In Figure 7a, blood glucose levels in HFD/STZ-induced MASLD mice peaked sharply at 30 min post-glucose administration, showing a significant increase compared to baseline (0 min). Although a progressive decline was observed thereafter, most notably between 30 and 120 min, there were no statistically significant differences in glucose levels between DCE-treated groups and the HFD/STZ group at any time, indicating that DCE had a limited impact on systemic glucose clearance. Despite the limited effect on glucose metabolism, hepatic glycogen content was notably affected by DCE treatment. The HFD/STZ group exhibited markedly reduced glycogen storage relative to controls. DCE administration, particularly at 200 mg/kg, significantly restored glycogen levels, suggesting improved hepatic glucose utilization and anabolic storage capacity (Figure 7b). This glycogenic effect was corroborated by Western blotting, which revealed a significant increase in the p-GSK3β/GSK3β ratio in the DCE-treated group (Figure 7c), indicative of glycogen synthase activation. These results suggest that DCE enhances hepatic glycogen synthesis independent of systemic glucose tolerance.

## 3. Discussion

The increasing prevalence of MSFLD in developed countries is probably attributable to the global rise in obesity and associated metabolic disorders [22]. Obesity, type 2 diabetes mellitus, and hypertriglyceridemia are well-established risk factors for MASLD [23]. MASLD is caused by abnormal fat accumulation in the liver, often indicative of insulin resistance, a common comorbidity in obese and diabetic individuals. While statins can reduce liver lipid deposition, their pharmacokinetic variability may raise concerns regarding potential hepatotoxicity [24]. Likewise, metformin, a frontline anti-diabetic agent, can improve hepatic gluconeogenesis and peripheral fatty acid oxidation, but is associated with gastrointestinal adverse effects [25]. These limitations have prompted growing interest in identifying safe, naturally derived compounds that target hepatic lipid metabolism.

*Desmodium caudatum* (Thunb.) DC. has been traditionally used in herbal medicine, and its bioactive constituents (including prenylated flavonoids and flavonols) have demonstrated antimicrobial, anti-inflammatory, and antioxidant activities [26,27]. In our study, treatment with DCE in the OA-induced hepatocyte model significantly improved cell viability, decreased ROS levels, and attenuated intracellular lipid accumulation (Figure 1 and Figure 2). Mechanistically, DCE regulated liver lipid metabolism by inhibiting triglyceride (SREBP-1, ACC, and FAS) and cholesterol (SREBP-2, HMGCR, and LDLR) synthesis, while simultaneously enhancing fatty acid oxidation through the upregulation of PPARα and CPT-1, potentially mediated by AMPK phosphorylation (Figure 3a–c). These concerted actions contribute to the observed reduction in hepatic lipid accumulation and align with previous reports on natural compounds such as oxymatrine, which improve lipid metabolism via similar regulatory mechanisms [28].

In addition to its lipid-modulating effects, DCE was found to restore glycogen content in OA-induced HepG2 cells (Figure 4a). This may be attributed to isovitexin and swertisin, two key flavonoids in DCE [29], which have been shown to enhance glycogen synthesis through the IRS-1/PI3K/AKT signaling pathway [30,31]. Our data confirmed that DCE increases the expression of IRS-1, AKT, and GSK3β (Figure 4b,c), key proteins involved in insulin-stimulated glycogen synthesis.

Consistent with our in vitro observations, DCE administration in a MASLD mouse model improved systemic metabolic parameters, including reductions in serum triglycerides, cholesterol, glucose, and liver injury markers (Table 1). Histological analysis demonstrated improvements in hepatic architecture and reduced steatosis (Figure 5 and Figure 6). Moreover, hepatic glycogen storage and synthesis were enhanced (Figure 7), supporting that DCE ameliorates lipid and glucose dysregulation in vivo. In related species, *Desmodium gangeticum* has been reported to exert hepatoprotective antioxidant effects by enhancing SOD, CAT, and GPx activities, while reducing lipid peroxidation (LPO) [32]. Similarly, *Prunus domestica* L. subsp. *syriaca* extract was shown to decrease ROS production, enhance glucose uptake, reduce lipid accumulation, and downregulate lipogenic genes such as DGAT1 and FASN in OA-induced HepG2 cells [33]. In addition, *Coriandrum sativum* L. leaf extract ameliorated hepatic steatosis in HFD-induced MASLD mice by attenuating body weight gain and liver/body weight ratio, while suppressing lipid droplet accumulation through the inhibition of lipid metabolism genes and the AMPK–SREBP1c pathway [34]. Likewise, *Coreopsis tinctoria* Nutt. extract reduced weight gain, hepatic lipid deposition, and improved dyslipidemia, inflammation, oxidative stress, and insulin resistance in HFD-fed mice via the regulation of the NF-κB/iNOS/COX-2/NLRP3/MAPK pathway [35]. Comparable to these reports, DCE also exerts antioxidant effects and reduces hepatic lipid accumulation. However, the added value of the present study lies in its comprehensive investigation of three distinct lipid metabolic pathways (lipogenesis, cholesterol metabolism, and lipid peroxidation) combined with in vivo comparisons against clinical drugs (simvastatin and metformin), which provided insights into different therapeutic strategies. Furthermore, the dual in vitro and in vivo validation further strengthens the significance of our findings. Interestingly, isovitexin has also been shown to suppress hepatic gluconeogenesis by downregulating FOXO1, PEPCK, and Glc-6-Pase [30], suggesting that DCE may exert broader regulatory effects on glucose metabolism, though this remains to be explored. Although DCE demonstrated apparent biological effects, the specific contributions of its bioactive compounds, such as isovitexin and swertisin, were not independently assessed. Further studies are warranted to clarify these components’ roles and to evaluate DCE’s clinical applicability and long-term safety.

## 4. Materials and Methods

### 4.1. Desmodium caudatum (Thunb.) DC. Extract (DCE)

*Desmodium caudatum* (Thunb.) DC. (Herbarium voucher number: TAIE051561, Taiwan Biodiversity Research Institute) was sourced from Taichung City. The identity of the plant material was confirmed through HPLC-ESI-MS/MS analysis, as detailed in our prior work [29]. For this study, the DCE was dissolved in a solution of 50% ethanol in deionized water to the desired experimental concentrations.

### 4.2. Cell Line and Treatment

The human hepatoma cell line HepG2 was obtained from the Bioresource Collection and Research Center (BCRC, Food Industry Research and Development Institute, Hsinchu, Taiwan). HepG2 cells were cultured in Eagle’s Minimum Essential Medium supplemented with 10% fetal bovine serum and 1% streptomycin, maintained at 37 °C in a humidified atmosphere containing 5% CO2. For experiments, HepG2 cells were seeded at a density of 6 × 10^4^ cells/mL in 6 cm dishes and allowed to adhere before any treatment.

### 4.3. Cell Viability Assay

To assess the potential cytotoxicity of DCE on HepG2 cells, cells were plated in a 6 cm dish with 6 × 10^4^ cells/mL per dish. Firstly, cells were exposed to varying concentrations of DCE (0, 25, 50, 100, 250, and 500 µg/mL) for 24 h. Subsequently, cells were detached using trypsin-EDTA, stained with propidium iodide, and analyzed using a Muse™ Cell Analyzer (EMD Millipore Corporation, Merck Life Sciences, KGaA, Darmstadt, Germany) to determine cell viability. In a separate experiment, the protective effect of DCE against oleic acid-induced cytotoxicity was evaluated. HepG2 cells were co-treated for 24 h with oleic acid (600 μM) in the presence or absence of DCE at 25, 50, and 100 μg/mL concentrations. Cell viability was then assessed using the Muse™ Cell Analyzer following PI staining.

### 4.4. Assessment of Intracellular ROS

Intracellular ROS levels were quantified using the fluorescent probe 2’,7’-dichlorofluorescein diacetate (DCF-DA). HepG2 cells were seeded in 6 cm dishes and incubated with DCF-DA diluted in culture medium for 1 h. Following this, cells were treated with or without DCE (25, 50, and 100 μg/mL) and oleic acid (600 μM) for 24 h. After treatment, cells were harvested, and the fluorescence intensity was measured using Muse™ Cell Analyzer.

### 4.5. Thiobarbituric Acid Reactive Substances (TBARS) Assay

Lipid peroxidation was assessed by measuring TBARS, which primarily reflect the malondialdehyde (MDA) levels and other aldehydes generated during lipid peroxidation. Cell lysates were collected by centrifugation and reacted with thiobarbituric acid (TBA) under heated acidic conditions. This reaction produces a pink chromophore with a maximum absorbance at 532 nm, which was quantified by a Multi-Mode Microplate Reader (Molecular Devices, San Jose, CA, USA). TBARS levels were expressed as MDA equivalents and normalized to the total cellular protein content of the corresponding samples.

### 4.6. Oil Red O Staining

A stock solution of Oil red O (Sigma-Aldrich, St. Louis, MO, USA) was prepared by dissolving the powder in 100% isopropanol (5 mg/mL) overnight. For in vitro staining, HepG2 cells were fixed in 10% formalin for 30 min. After that, cells were stained with a working solution of Oil red O, which was diluted with deionized water. After staining, cells were rinsed to remove excess stain. Images of the stained cells were captured using a light microscope. The Oil red O stain was extracted from the cells by adding 100% isopropanol to quantify intracellular lipid accumulation. The extract’s absorbance was measured at 492 nm using a Multi-Mode Microplate Reader. For in vivo analysis, frozen liver tissue sections were stained with the working Oil red O solution. After staining, sections were rinsed with deionized water for 15 min and then mounted using glycerol–gelatin (Sigma-Aldrich, St. Louis, MO, USA). The stained tissue sections were observed under a light microscope, and the extent of lipid accumulation was quantified using ImageJ software (v1.51k).

### 4.7. Nile Red Staining

Total intracellular lipid content was evaluated using Nile red staining. A stock of Nile red (Sigma-Aldrich, St. Louis, MO, USA) was dissolved in acetone and diluted in deionized water as a working solution. In vitro, HepG2 cells were fixed with 4% paraformaldehyde for 30 min and stained with the Nile red working solution for 5 min. Following staining, the fluorescence intensity was measured using the Muse™ Cell Analyzer with excitation at 480 nm. For in vivo, fresh-frozen tissue sections were stained with the Nile red working solution for 5 min. The stained sections were examined using fluorescence microscopy with excitation/emission wavelengths set at 470/590 nm. Quantification of lipid content in the tissue sections was performed using ImageJ software.

### 4.8. Animal and Experimental Design

Six-week-old male C57BL/6 mice were obtained from Bio LASCO Taiwan Co., Ltd. (Taipei, Taiwan). All animal procedures were conducted according to the guidelines of the Institutional Animal Care and Use Committee (IACUC, Taipei, Taiwan) of Chung Shan Medical University (license number 2426). Animals were housed under controlled conditions of temperature and humidity and with a 12 h light/dark cycle and allowed free access to food and water. Mice were randomly divided into seven groups as follows: (1) Control group (Chow diet) (n = 10), (2) HFD/STZ group (n = 10), (3) HFD/STZ +DCE100 (100 mg/kg bw) group (n = 10), (4) HFD/STZ +DCE200 (200 mg/kg bw) group (n = 10), (5) HFD/STZ +Simvastatin (200 mg/kg bw) group (n = 10), (6) HFD/STZ +Metformin (250 mg/kg bw) group (n = 10), and (7) DCE-only (200 mg/kg bw) group (n = 6). Mice in the control and DCE-only groups received a standard commercial chow diet (Laboratory Rodent Diet-5001, LRD-5001), while all other groups were fed a high-fat diet (HFD; 60% kcal from fat, DIO rodent diet 58Y1, Test Diet^®^, Richmond, IN, USA) for 12 weeks to induce MASLD. To further replicate metabolic syndrome features, mice in the HFD-fed groups received intraperitoneal injections of streptozotocin (STZ, 40 mg/kg bw) for five consecutive days during week 4. The establishment of MASLD was confirmed in week 5 using an oral glucose tolerance test (OGTT) and measurement of fasting serum glucose. Starting from week 6, mice in the treatment groups received daily oral gavage with either *Desmodium caudatum* extract (DCE, 100 or 200 mg/kg), Simvastatin (200 mg/kg), or Metformin (250 mg/kg) for six weeks. Simvastatin, a lipid-lowering agent, is clinically used for hypercholesterolemia [36]. Metformin, an insulin sensitizer, is commonly prescribed for type 2 diabetes mellitus to reduce hepatic glucose production and improve peripheral glucose uptake [37]. All compounds were dissolved in sterile saline before administration. After 12 weeks, all mice were sacrificed, and serum and liver tissue samples were collected for subsequent analyses.

### 4.9. Oral Glucose Tolerance Test (OGTT)

An OGTT was performed after a 12 h fasting period. A glucose solution (2 g glucose/kg bw) was prepared in saline and administered to the mice via oral gavage. Blood samples were collected from the tail vein at specific time points: 0, 30, 90, and 120 min post-gavage. Serum glucose concentrations were then measured from these collected samples to assess glucose tolerance.

### 4.10. Histopathological Examination

Following sacrifice, liver tissues from all animals were fixed in ethanol solutions and subsequently embedded in paraffin blocks. Sections with a thickness of 3 μm were cut from these paraffin blocks and mounted onto glass slides. The tissue sections were stained using a Hematoxylin–Eosin (H&E) Staining Kit (BioVision, Milpitas, CA, USA). Histological changes were examined under a light microscope, and the extent of these changes was quantified using ImageJ software.

### 4.11. Western Blotting

Total proteins were extracted from HepG2 cells and liver tissue samples by homogenization in 300 μL of RIPA lysis buffer supplemented with a protease inhibitor cocktail (Pierce; Thermo Fisher Scientific, Waltham, MA, USA). Equal amounts of protein from each sample were separated by sodium dodecyl sulfate-polyacrylamide gel electrophoresis and subsequently transferred onto nitrocellulose membranes (Millipore, Bedford, MA, USA). To minimize non-specific antibody binding, the membranes were blocked by incubation in 5% nonfat milk in Tris-buffered saline with 0.1% Tween-20 for 1 h at room temperature. The membranes were then incubated overnight at 4 °C with the following primary antibodies: SREBP-1 (sc-13551), SREBP-2C (sc-13552), HMGCR (sc-271595), LDLR (sc-11824), ACCα (sc-30212), p-ACCα (sc-271965), FAS (sc-55580), PPARα (sc-9000), CPT-1 (sc-20669), AMPKα (sc-74461), IRS-1 (sc-560), GSK3β (sc-9166), and p-IRS-1 (sc-33956), purchased from Santa Cruz Biotechnology (Paso Robles, CA, USA). The primary antibodies p-AMPKα (2535s), p-AKT (4051s), and p-GSK3β (9336) were purchased from Cell Signaling Technology (Boston, MA, USA). A primary antibody pan-AKT1/2/3 (AF6261) was sourced from Affinity Biosciences (Buckingham, United Kingdom, Europe). β-actin (A5441), used as an internal loading control, was purchased from Sigma-Aldrich (St Louis, MO, USA). Following primary antibody incubation, the samples were incubated with the secondary antibodies for 1 h at 4 °C. Finally, the protein bands were visualized using an enhanced chemiluminescence detection system, and the band intensities were quantified using an ImageQuant™ LAS 4000 mini (GE Healthcare Bio-Sciences AB, Uppsala, Sweden). Protein expression levels were normalized to the intensity of the β-actin loading control.

### 4.12. Glycogen Assay

Glycogen content in HepG2 cells and liver tissue samples was determined using the EnzyChrom™ Glycogen Assay Kit (BioAssay Systems, Hayward, CA, USA). Lysed cells, homogenized tissue samples, and glycogen standards provided in the kit were treated with the working reagent to hydrolyze glycogen into glucose. The liberated glucose then reacted with a specific probe in a coupled enzymatic assay to generate a colored product. Following a 30 min incubation in the dark at room temperature, the absorbance of the resulting solution was measured at 570 nm using a microplate reader. Glycogen concentrations in the samples (μg/mL or μg/mg tissue) were calculated based on the standard curve generated from the provided glycogen standards.

### 4.13. Statistical Analysis

All data are presented as the mean ± standard deviation (SD) derived from at least three independent experiments. For comparisons between two groups involving a single independent variable, statistical significance was assessed using Student’s *t*-test. For comparisons across multiple treatment groups, a one-way analysis of variance (ANOVA) was performed to determine significant differences relative to the untreated control group. In analyses involving repeated measurements within the same subjects, such as blood glucose levels at different time points in the OGTT, a paired *t*-test was employed to evaluate intra-group temporal differences. A *p*-value of less than 0.05 (*p* < 0.05) was considered statistically significant. All statistical analyses were conducted using Sigma Plot 10.0 software (Systat Software, Inc., San Jose, CA, USA).

## 5. Conclusions

Our findings demonstrate that DCE exerts robust hepatoprotective effects against MASLD via multifaceted mechanisms. By activating AMPK signaling, DCE suppresses de novo lipogenesis and enhances fatty acid oxidation, while concurrently stimulating glycogen synthesis through the AKT/GSK3β pathway. These integrated metabolic effects contribute to improved hepatic lipid and glucose homeostasis. Taken together, the evidence positions DCE as a promising therapeutic candidate for MASLD with both hypolipidemic and hypoglycemic properties. Future studies should investigate its long-term efficacy, potential synergy with existing pharmacotherapies, and translational value in clinical applications.

## Figures and Tables

**Table 1:** Effect of DCE on the serum biochemical parameters in HFD/STZ-induced MASLD mice.

	Control	HFD/STZ	HFD/STZ +D100	HFD/STZ +D200	HFD/STZ +Simvastatin	HFD/STZ +Metformin	D200
GLC (mg/dL)	104.29 ± 10.23	291.86 ± 38.51 ^##^	164.83 ± 31.74 **	269.33 ± 66.40	303.50 ± 54.47	181.20 ± 51.63 **	86.20 ± 28.60
TC (mg/dL)	81.00 ± 17.51	178.00 ± 7.94 ^##^	144.50 ± 20.11 *	122.40 ± 27.53 *	161.33 ± 4.73 *	124.60 ± 30.86 *	82.33 ± 4.04
TG (mg/dL)	65.00 ± 8.75	140.00 ± 8.54 ^##^	117.00 ± 11.53	102.33 ± 6.11 **	154.00 ± 21.21	112.75 ± 22.20 *	59.00 ± 13.23 ^##^
LDL-C (mg/dL)	5.80 ± 0.75	7.87 ± 1.08 ^#^	4.75 ± 1.32 *	3.68 ± 1.44 **	6.85 ± 2.67	4.72 ± 1.80	2.33 ± 0.21
HDL-C (mg/dL)	59.60 ± 2.78	40.90 ± 0.71 ^##^	76.95 ± 5.93 **	65.03 ± 5.87 **	73.76 ± 5.24 **	68.30 ± 2.60 **	53.83 ± 4.63
AST (U/L)	176.33 ± 7.09	246.33 ± 29.48 ^#^	171.00 ± 20.54 *	138.50 ± 30.95 *	166.67 ± 9.24 *	148.00 ± 31.18 *	148.00 ± 11.22 ^#^
ALT (U/L)	29.80 ± 2.95	45.67 ± 7.41 ^##^	31.67 ± 6.34 *	33.00 ± 4.97 *	28.00 ± 8.12 *	40.67 ± 8.39	30.67 ± 3.79
LDH (U/L)	882.33 ± 156.05	1830.67 ± 579.71 ^#^	745.00 ± 65.65 **	697.50 ± 144.34 **	687.00 ± 38.11 **	676.29 ± 106.39 **	685.33 ± 158.45 ^#^

Data are presented as the mean ± SD (n = 3). Statistical significance was assessed using Student’s *t*-test. ^#^ *p* < 0.05, ^##^ *p* < 0.01 compared with the control group. * *p* < 0.05, ** *p* < 0.01 compared with the HFD/STZ-induced group. HS and HFD combine with STZ to induce MASLD. D100, 100 mg/kg bw *Desmodium caudatum* (Thunb.) DC. extract. D200, 200 mg/kg bw *Desmodium caudatum* (Thunb.) DC. extract. GLC, fasting blood glucose. TC, total cholesterol. TG, triglyceride. LDL-C, low-density lipoprotein-cholesterol. HDL-C, high-density lipoprotein-cholesterol. AST, aspartate aminotransferase. ALT, alanine aminotransferase. LDH, lactate dehydrogenase.

## Data Availability

The complete dataset is available upon request from the authors.

## Supplementary Materials

The following supporting information can be downloaded at https://www.mdpi.com/article/10.3390/ijms26178442/s1.

## Abbreviations

The following abbreviations are used in this manuscript:

ACC	Acetyl-CoA carboxylase
AKT	Serine/threonine kinase (protein kinase B)
ALD	Alcoholic liver disease
ALT	Alanine aminotransferase
AMPK	AMP-activated protein kinase
AST	Aspartate aminotransferase
CPT-1	Carnitine palmitoyltransferase I
DCE	*Desmodium caudatum* (Thunb.) DC. extract
DCF-DA	2’,7’-dichlorofluorescein diacetate
FAS	Fatty acid synthase
GLC	Fasting blood glucose
GS	Glycogen synthase
GSK3β	Glycogen synthase kinase 3 beta
HDL-C	High-density lipoprotein-cholesterol
HFD	High-fat diet
HMGCR	Hydroxymethylglutaryl-CoA reductase
IRS-1	Insulin receptor substrate 1
IRS-2	Insulin receptor substrate 2
LDH	Lactate dehydrogenase
LDL-C	Low-density lipoprotein-cholesterol
LDLR	Low-density lipoprotein receptor
MASH	Metabolic dysfunction-associated steatohepatitis
MASLD	Metabolic dysfunction-associated steatotic liver disease
MDA	Malondialdehyde
OA	Oleic acid
OGTT	Oral glucose tolerance test
PI3K	Phosphoinositide 3-kinase
PPARα	Peroxisome proliferator-activated receptor α
ROS	Reactive oxygen species
Ser	Serine
SREBP-2	Sterol regulatory element-binding protein 2
STZ	Streptozotocin
TBARS	Thiobarbituric acid reactive substances
TC	Total cholesterol
TCM	Traditional Chinese Medicine
TG	Triglyceride
Tyr	Tyrosine
