# Supplementary material for: A Plant-Based Strategy for MASLD: Desmodium caudatum (Thunb.) DC. Extract Reduces Hepatic Lipid Accumulation and Improves Glycogen Storage In Vitro and In Vivo
Source: Int J Mol Sci. 2025 Aug 30;26(17):8442. doi: 10.3390/ijms26178442 (PMC12429059; doi:10.3390/ijms26178442)
Supplement: Supplementary file 1 [file ijms-26-08442-s001.zip › ijms-3819224-supplementary.pdf]

Figure S1

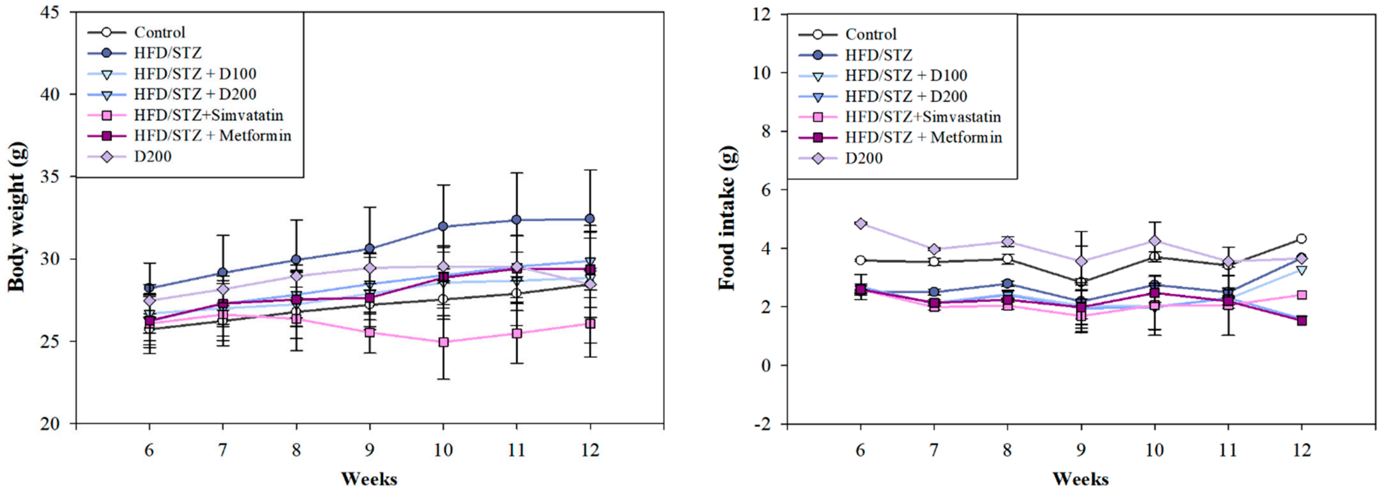

(a) **(b)**

Figure S1. Effects of DCE on body weight and food intake in HFD/STZ-induced MASLD mice. MASLD mice were induced by HFD combined with STZ injection. At the 6th week, mice were treated with DCE (100 and 200 mg /kg bw), Simvastatin (200 mg/kg bw), and Metformin (250 mg/kg bw) for 6 weeks. The mice were sacrificed after 6 weeks, and liver tissue was collected for analysis. The body weight **(a)** and food intake **(b)** were recorded during 6 weeks. Quantitative data are presented as mean ± SD (n=3). $^{\#}p < 0.05$, $^{\#\#}p < 0.01$ compared with the control group. $^{*}p < 0.05$, $^{**}p < 0.01$ compared with the HFD/STZ-induced group. HFD/STZ and HFD combined with STZ induce MASLD. D100, 100mg/kg bw *Desmodium caudatum (Thunb.)* DC. Extract. D200, 200mg/kg bw *Desmodium caudatum (Thunb.)* DC. Extract. OGTT, Oral glucose tolerance test.
